# Evidence-based recommendation of a powered knee for transfemoral bone-anchored prostheses: A cross-sectional study

**DOI:** 10.33137/cpoj.v8i2.45790

**Published:** 2025-11-02

**Authors:** L Frossard, S Laux, M Geada, L Tronicke, T Fridriksson, K Lechler

**Affiliations:** 1 YourResearchProject Pty Ltd, Brisbane, Australia.; 2 Griffith University, Southport, Australia.; 3 Queensland University of Technology, Brisbane, Australia.; 4 University of the Sunshine Coast, Sippy Downs, Australia.; 5 APC Prosthetics Pty Ltd, Alexandria, Australia.; 6 ÖSSUR, R&D, Medical Office, Reykjavik, Iceland.

**Keywords:** Amputation, Artificial Limbs, Bionics, Kinetics, Loading, Bone-Anchored Prosthesis, Lower Limb, Prosthesis, Osseointegrated Implant, Microprocessor-Controlled Knees

## Abstract

**BACKGROUND:**

A transfemoral bone-anchored prosthesis (TF-BAP) can be fitted with non-microprocessor-controlled knees (N-MPKs), or with microprocessor-controlled knees, which can be passive (P-MPKs) or active (A-MPKs). The next generation of A-MPKs, including powered knees, is emerging. The understanding of the loading applied on TF-BAP fitted with these A-MPKs is limited.

**OBJECTIVE:**

This cross-sectional study aimed to characterize the load applied on instrumented TF-BAP fitted with an A-MPK (Power Knee, Össur, Iceland) during standardized daily activities. Furthermore, some load characteristics applied during walking were compared with TF-BAP fitted with N-MPK and P-MPK reported in the literature using similar approach.

**METHODOLOGY:**

Thirteen males fitted with a transfemoral press-fit osseointegrated implant participated in this study between 2021 and 2022. Forces and moments applied on the instrumented TF-BAP, fitted with a Power Knee (PKA01) and Pro-Flex (LP, XC) or Balance S feet (ÖSSUR, Iceland), were measured wirelessly using an iPecsLab (RTC Electronics, USA) during walking, ascending and descending ramp and stairs. We followed a 28-step process to characterize the loading pattern considering spatiotemporal gaits variables as well as loading boundaries and extrema.

**FINDINGS:**

Overall, 1,327 steps were analyzed. The cadence ranged between 34 ± 6 and 49 ± 13 strides/min. The maximum forces and moments recorded on the long, anteroposterior and mediolateral axes of the transducer were 1,258 N, 331 N and 234 N as well as 19 Nm, 74 Nm and 91 Nm, respectively.

**CONCLUSION:**

The Power Knee, combined with Pro-Flex or Balance S feet, may improve participants' capacity to ambulate. Comparations with reference values indicated that transitions from N-MPKs or P-MPKs to the Power Knee are considered safe and likely to improve efficiency. This study contributed to evidence-based recommendations of TF-BAP fitted with powered knees. Hopefully, this work will advance clinical practice guidelines for the growing population choosing bionic solutions.

## INTRODUCTION

### Clinical Outcomes of Bone-Anchored Prostheses

**Figure S1:**
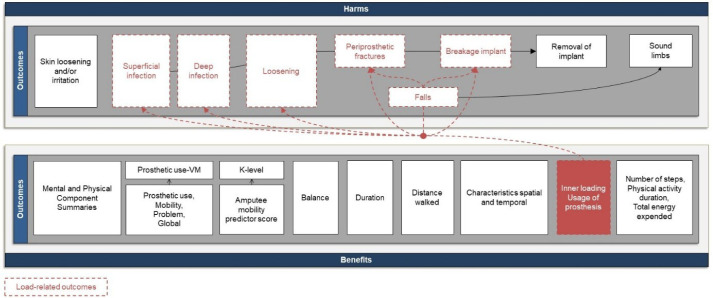
Overview of six load-related outcomes susceptible to have a cause-effect relationship between the usage of the bone-anchored prosthesis (e.g., onset, magnitude and duration of loading) and the nine most frequent adverse events. Adapted from reference 4, and 5.

A bone-anchored prosthesis (BAP) connected to an osseointegrated implant might be indicated for some individuals with transfemoral amputation (TFA).^1-3^ In principle, daily loading regimen applied on the implant during prosthesis use may be related to at least six of the nine most common transfemoral bone-anchored prosthesis (TF-BAP) adverse events (e.g., superficial and deep infections, loosening, falls, periprosthetic fractures and implant breakage; **Appendix-Figure S1**).^4,5^ Ideally, the loads applied during rehabilitation and beyond should be within a pain-free and bespoke “Goldilocks zone” where the “right load” is applied at the “right time”.^6-8^ In all cases, it is critical to understand how the loading profiles vary between TF-BAP equipped with compatible components.^9-12^

### Understanding Prosthetic Loading Profile

Approximately 65 publications referred to loading characteristics associated with lower limb BAPs, including landmark reviews presented by Niswander et al (2020) and Ravari et al (2024).^13,14^ These reviews indicated that portable kinetic systems including tri-axial transducers embedded into a lower limb BAPs have been used to measure directly the load profile applied on osseointegrated implants during standardized rehabilitation and daily activities.^6,10,11,15-24^

Cross-sectional cohort studies have previously looked at instrumented TF-BAP fitted with components recommended as best-practice at the time, such as Total Knee 1900, C-Leg and Rheo Knee XC.^6,15,16,22-24^ In our previous study, 10 TFAs were fitted mainly with basic prosthetic knees components such as mechanically passive knees or non-microprocessor-controlled knee (N-MPKs), the first passive microprocessor-controlled knee (P-MPK) and various prosthetic feet component.^15,16,25^ More recently, in our study, 13 TFAs were fitted with the Rheo Knee XC (Össur) and energy-storing-and-returning feet (ESARs).^6,26^ As presented in Frossard et al,^25,26^ the maximum force and moment expressed as a percentage of body weight (%BW) were 84 ± 7 %BW and 2.63 ± 1.04 %BW·m while walking with a TF-BAP fitted with the GaitMaster, Total Knee 1900, Adaptive, or C-Leg, and 102 ± 7 %BW and 3.44 ± 0.98 %BW·m while walking with the Rheo Knee XC. Over time, the repetition of such studies has led to a more standardized ecological approach to characterize loading profiles. The strength of this ecological approach is to report everyday loading regimen but its limitation is to overlook informative 3D dynamic, kinematic and inverse dynamic data.^17,21,27-32^

### Need for Characterization of Loading Profile with the Power Knee

Nowadays, the prescription of recent P-MPKs and ESARs components is commonly recognized as the cost-effective standard of care when fitting TF-BAP.^1,6,13,14,33-37^ The next generation of active microprocessor-controlled knees (A-MPKs), including powered knees, is emerging.^38-40^ A-MPKs use motors to actively assist with activities such as walking, standing up and climbing stairs. The development of active prostheses is the subject of many research projects with the vision of making walking with a prosthesis more natural and efficient for the user through adaptive actuators.^39-42^ The Power Knee (Össur, Iceland) is the first commercially available motor-powered A-MPK. It actively supports flexion and extension adapted to the user's activity.^43^ Furthermore, it can improve the loading symmetry between sound and prosthetic sides during walking and sit-to-stand movements and supports the user during step-over-step stair ascent.^44^

To the best of our knowledge, there are limited studies on the loading profile associated with TF-BAP fitted with the Power Knee.^36^ Therefore, there is a need for studies characterizing the loading profile using an ecological approach to facilitate comparisons with previous studies that focused on other recommended components for TF-BAP.

### Purposes

The purpose of this cross-sectional study was to characterize the loading profile applied on TF-BAP when fitted with the Power Knee using an ecological approach (e.g., direct measurement of the load in quasi real-world conditions). The specific objectives were to:

1. Present the range and variability of spatiotemporal gait variables, the magnitude of loading boundaries, as well as the onset and magnitude of extrema applied to the instrumented TF-BAP fitted with a Power Knee, Pro-Flex (LP or XC), or Balance S feet during standardized straight-level walking and while ascending and descending ramps and stairs,
2. Compare some of these loading characteristics with those reported in the literature for TF-BAPs fitted with N-MPKs and a recent P-MPK (Rheo Knee XC).^6,15,16,20,25,26^

## METHODOLOGY

### Design

This cross-sectional interventional study was a part of a registered clinical trial (ClinicalTrials.gov: 2021-YRP-LLA-Load-01).

### Populations

Individuals fitted with TF-BAP were recruited by a prosthetist using established selection criteria presented in **Appendix-Table S1**.^6,15,16,20,26,45^ There was no specific exclusion criteria related to gender, ethnicity, height or functional level. All participants were fitted with a non-FDA approved press-fit implant, (i.e., Osseointegration Prosthetic Limb, Permedica SPA, Italy). Assessment took place in Sydney, Australia between December 2021 and June 2022. Participants signed a written ethical consent form (Protocol No: Bellberry HREC-2021-YRP-LLA-Load-01).

### Extraction

Load profile was extracted following a standardized 28-step process outlined in **Table 1** that emerged from previous studies.^6,10,11,15,16,18,20,22,26^ Key steps of the process are detailed below.

**Table 1:** Overview of the standard ecological approach relying on 28-step process to record, extract and analyze to load profile applied on bone-anchored prosthesis emerging from the literature. **F:** Force; **M:** moment; **LG:** Long axis; **AP:** Anteroposterior axis; **ML:** Mediolateral axis; **GC:** Gait cycle; **%GC:** Percentage of gait cycle; **SUP:** Support phase; **%SUP:** Percentage of support phase; **%BW:** Percentage of bodyweight; **%BWm:** Percentage of bodyweight per meter; **FLG1:** First point of interest of the force applied on the long axis; **FAP1:** First point of interest of the force applied on the anteroposterior axis; **FML1:** First point of interest of the force applied on the mediolateral axis; **MLG1:** First point of interest of the moment applied around on the long axis; **MAP1:** First point of interest of the moment applied around the anteroposterior axis; **MML1:** First point of interest of the moment applied around the mediolateral axis; **FAP2:** Second point of interest of the force applied on the anteroposterior axis; **MLG2:** Second point of interest of the moment applied around on the long axis; **MML2:** Second point of interest of the moment applied around the mediolateral axis; **MML3:** Third point of interest of the moment applied around mediolateral axis.

Step	Description
**A-Recording A.1-Equipment**	
**A.1.1-Portable kinetic system (iPecsLab, RTC Electronics, USA)**	
1	Setup sampling recording of forces and moments at 200 Hz
2	Setup connection so that loading data are sent wirelessly to laptop nearby
3	Ensure that the forces and moments were measured with an accuracy of ±0.01 N and ±0.001 Nm, respectively
4	Fit transducer of the iPecsLab to the instrumented prosthesis
5	Align the coordinate system of the transducer that its vertical axis was co-axial with the long (LG) axis of the implant and the other axes corresponded to the anatomical anteroposterior (AP) and mediolateral (ML) directions of the implant
6	Denote forces acting on the three axes of the transducer as FLG, FAP and FML where compression, anterior and lateral forces were positive, respectively
7	Denote moments around the three axes of the transducer as MLG, MAP and MML where external, lateral and anterior moments were positive, respectively
8	A prosthetist performed a standard static alignment of the prosthesis guided by principles outlined in the literature
9	Perform dynamic alignment and resistance adjustment for knee and foot that suited participants' preferences and comfort
10	Consider that the medullar and percutaneous parts of the implant as well as the tube and/or adaptor were one rigid part
11	Measure the offset of the distal end of connector attached to the percutaneous part and the centre of the Power Knee in relation to the origin of coordinate system of iPecsLab's transducer
12	Calibrate the transducer at the end of the recording session when the prosthesis was removed using post recording bench top measurements (i.e., zero-offset)
**A.1.2-Video recording**	
13	Setup basic video recording using digital camera of each trial of daily activities to facilitate the analyzes and interpretation of the loading data (e.g., digital notebook)
**A.2-Activities**	
14	Measure characteristics of physical setup used to perform straight level walking as well as ascending and descending ramp and stairs
15	Train participant on how to use the Power Knee functions prior each of the ascending and descending ramp and stairs activities
16	Ask participants to perform up to five trials in each activity consecutively at a self-selected comfortable pace and to use the handrail, if needed
17	Advise participants to use the step-over-step (e.g., normal reciprocal stepping pattern) rather that step-by-step (e.g., placement of both feet on the same step before the next step) technique while ascending and descending stairs, when possible
18	Acclimate and practice with instrumented prosthesis for 30–60 minutes prior each activity
**B-Processing**	
19	Calibrate the raw forces and moments for each trial by considering the magnitude of the load recorded during calibration
20	Detect of relevant segment of loading data by discarding the first and the last two to three strides recorded for each trial so that the steps analyzed where at a steady pace, outside of gait initiation and termination, respectively
21	Determine of gait events using the plot of FLG to detect manually individual heel contacts and toe-offs events within the relevant segment for each trial
22	Normalize datasets by the time from 0 to 100 throughout the gait cycle (GC) or support phases (SUP) to facilitate averaging of trials as well as reporting of spatiotemporal characteristic and extrema in percentage of gait cycle (%GC) or support (%SUP), respectively
23	Normalize forces and moments datasets by percentage of bodyweight (%BW, %BWm)
**C-Analysis**	
24	Extract three spatiotemporal variables including the cadence in strides per minute (stride/min) for a given trial (i.e., duration between two consecutive heel contacts of the prosthetic limb so that cadence of prosthetic limb did not always equate to the number of steps ascended or descended during stairs activities depending on step-over-step or step-by-step technique), duration of gait cycle in seconds (s), and duration of the support phases in percentage of gait cycle (%GC)
25	Extract 12 loading boundaries across all gait cycles per activity regardless of the onset including the minimum, maximum, and maximum of the absolute minimum and maximum magnitude of forces in N and %BW and moments in Nm and %BWm
26	Extract 36 overall loading boundaries across all activities including the minimum, maximum, and maximum of the absolute minimum and maximum magnitude of forces in N and %BW and moments in Nm and %BWm
27	Extract semi-automatically (e.g., searching the minimum or maximum magnitude of forces and moment within a pre-set time window) up to 10 loading extrema (i.e., points of inflection of the loading pattern occurring consistently over successive steps for a given activity for all participants per activity including onset in %SUP (i.e., time of occurrence of extremum) and magnitude in N and %BW or Nm and %BWm (i.e., minimum or maximum magnitude of point on the curve of forces and moment within a pre-set time window)
28	Characterize weight acceptance and propelling loading considering six (i.e., FLG1, FAP1, FML1, MLG1, MAP1, MML1) and four (i.e., FAP2, MLG2, MML2, MML3) loading extrema occurring during the critical initial and final phases of the gait cycle, respectively

### Recording

**Figure S2:**
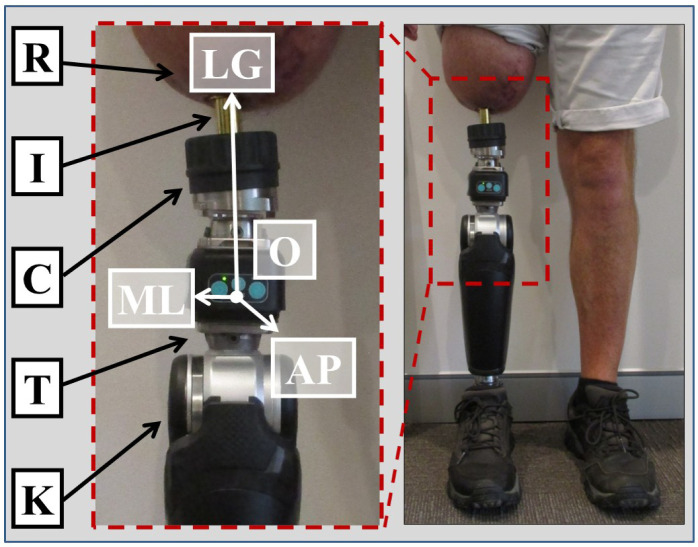
Example of instrumented transfemoral bone-anchored prostheses including position and orientation of tri-axial transducer coordinate system of the portable kinetic system (iPecsLab, RTC Electronics, USA) for Participant 1. **O:** Origin of the transducer coordinate system, **LG:** Long axis, **AP:** Antero-posterior axis, **ML:** Medio-lateral axis, **R:** Residuum, **I:** Percutaneous part of the implant, **C:** Connector, **T:** Transducer, **K:** prosthetic knee.

The loading was recorded during Step 1–18 (**Table 1-A**). The instrumented prostheses included iPecsLab's transducer (RTC Electronics, USA) fitted between the connector and a Power Knee (n = 13, 100%) so that loading could be measured directly (**Appendix-Figure S2**). The participants were fitted with Pro-Flex LP (n = 7, 53%), Pro-Flex XC (n = 4, 30%) or Balance S (n = 2, 15%) prosthetic feet and their own footwear. We purposely chose the LP and XC models within the Pro-Flex ankles family which are commonly recommended for patients in Australia based on their ability to tolerate high impacts.

**Figure S3:**
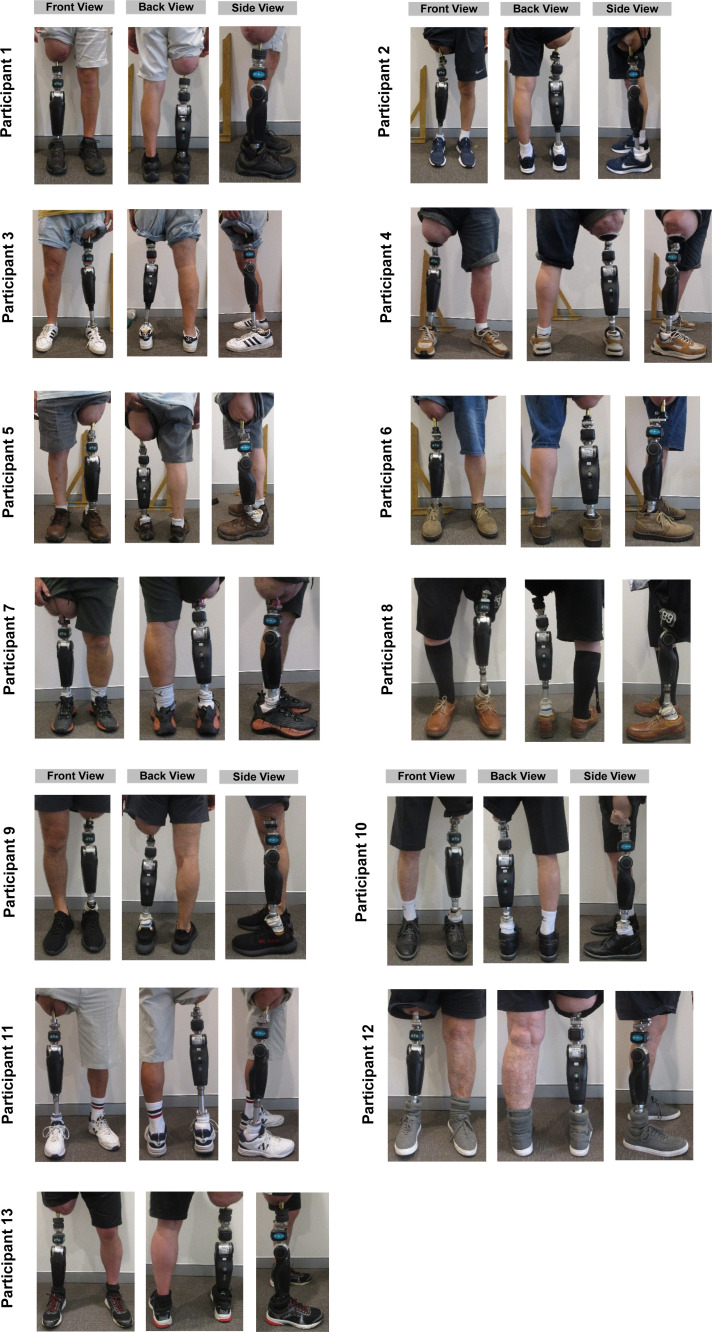
Individual alignment of the instrumented bone-anchored prosthesis fitted with iPecsLab's transducer (RTC Electronics, USA) instrumented prosthesis (i.e., Power Knee, Pro-Flex LP, Pro-Flex XC, Balance S) and footwear for the cohort of 13 participants.

**Figure S4:**
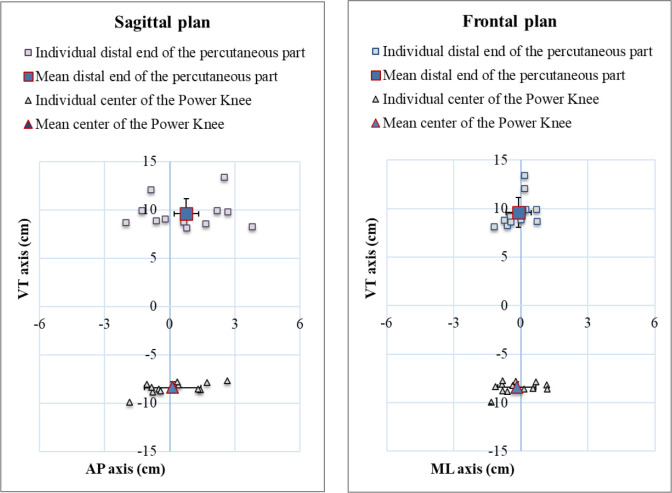
Position of the distal end of connector attached to the percutaneous part and the geometrical centre of the Power Knee in relation to the origin of coordinate system of iPecsLab's transducer (RTC Electronics, USA) on the antero-posterior (AP), medio-lateral (ML) and vertical (VT) axes of the sagittal and frontal planes.

**Figure S5:**
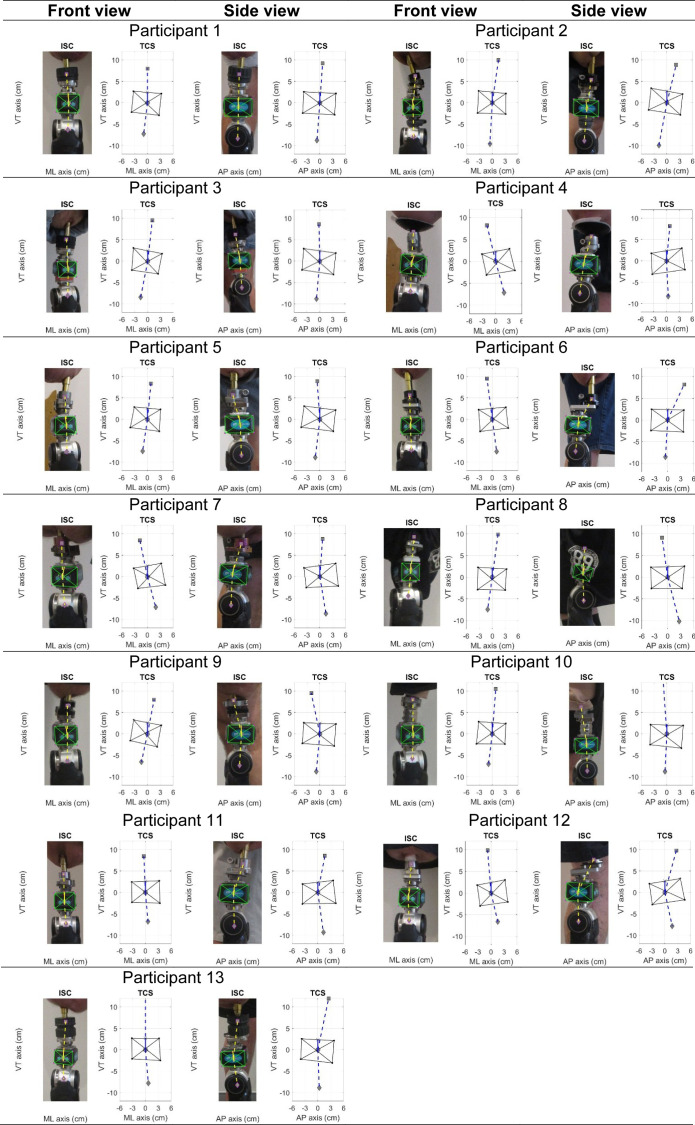
Individual position of the distal end of connector attached to the percutaneous part and the geometrical centre of the Power Knee in relation to the origin of iPecsLab's transducer (RTC Electronics, USA) on the antero-posterior (AP), medio-lateral (ML) and vertical (VT) axes of the front and side views in the image (ICS) and transducer (TCS) coordinate systems.

Each force (F) and moment (M) were measured wirelessly at 200 Hz and expressed in the transducer's coordinate system (**Appendix-Figure S2**). It was aligned so that its axes corresponded as closely as possible to the anatomical long (LG), anteroposterior (AP) and mediolateral (ML) axes of the implant (**Appendix-Figure S3**). A prosthetist performed a standard static alignment guided by principles outlined in the literature.^46^ In all instances, the co-linearity of the long axes of the implant and the transducer depended on the offset of the connector used to achieve the desired alignment (**Appendix-Table S2, Figure S4, Figure S5**). The prosthetist also performed a dynamic alignment and adjusted the knee settings that suited participants' preferences and comfort.

The loading was measured while participants performed successively up to five trials of straight level walking, ascending and descending ramp and stairs (**Appendix-Table S3**).

Participants were used to walk with a P-MPK such as C-Leg or Genium (Ottobock, USA) or Rheo Knee XC (Össur, Iceland) or the Power Knee. Regardless, they were trained on how to use the Power Knee functions prior each activity (e.g., step-over-step technique to ascend and descend stairs). Approximately 30–60 minutes of acclimation with the prosthesis were initially deemed sufficient to achieve the required confidence and warrant safety based on literature.^47^ Participants were instructed to perform each activity at a self-selected pace and to use the handrail if needed.

### Processing

The loading was processed during Step 19–23 (**Table 1-B**) using customized Matlab software program (The MathWorks Inc., USA)^6,15,16,20,25,26^ This program enabled the identification of gait events as well as time normalization over the percentage of a gait cycle (GC) or support phase (SUP) and normalization of loading datasets by percentage of bodyweight (%BW, %BWm).

### Analysis

The loading was analyzed during Step 24–28 (**Table 1-C**), also using Matlab software program. The loading profile was characterized using spatiotemporal variables, loading boundaries, and up to 10 loading extrema depending on the activities. For this study, we purposely characterized the loading during critical phases of GC (**Table 1-Step 28**), including:

- Weight acceptance using six extrema occurring during initial phasis of the GC where the bodyweight must be applied onto the knee smoothly for comfort and safely to action stance control features,
- Propelling loading using four extrema occurring during the final phasis of the GC where the knee should assist shifting the center of mass slightly sideway and more importantly forward onto the sound limb.

### Statistics

The mean and standard deviation of spatiotemporal variables, loading boundaries and extrema were calculated after collating all GCs recorded for each activity.

The variability of the dataset was determined using the percentage of variation (PV), calculated as:



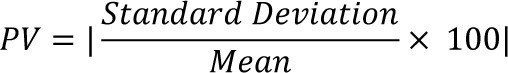


To be consistent with the literature reporting inter- and intra-subject variability of loading data, we considered a PV below 20% to indicate low variability and a PV above 20% to indicate high variability, respectively.^6,15,16,20,22,25,26^

### Comparisons

Selected indicators of the loading profile were benchmarked against reference datasets extracted from the literature including able-bodied participants as well as TFAs fitted with socket prostheses, N-MPKs (n = 8) and P-MPKs (n = 13).^6,15,16,20,25,26^

We only considered previous studies that used a similar protocol to reduce the confounding effects of the measurements (e.g., selection criteria, direct load measurement, loading characterization).

Differences between discrete indicators including spatiotemporal gait variables as well as loading boundaries and extrema were determined so that a positive difference indicated that the Power Knee was algebraically larger than the reference datasets. The relative difference between indicators was also expressed as a percentage of the Power Knee:



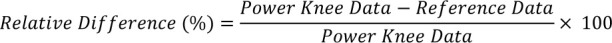


We considered that an absolute relative difference superior to 10% was above a minimal clinically important difference (MCID). This threshold might appear low compared to other studies considering an MCID of 20% when comparing prosthetic knee components.^50^ Conservatively, we believe that a lower MCID was justified in the particular case of individuals fitted with TF-BAP given that their proprioception is increased due to osseoperception provided by the implant.^6,51^

## RESULTS

A cohort of 13 males with TFAs participated in this study (64 ± 13 years; 1.79 ± 0.06 m; 93.7 ± 15.5 kg; 27.6 ± 4.2 kg/m^2^), as detailed in **Table 2**. Participation of only males was unintended and accidental. The surgical timeline was 11 ± 9 years since amputation and 6 ± 3 years since implantation. The residuum length was 33.6 ± 4.9 cm or 71 ± 10 % of sound thigh.

**Table 2:** Overall and individual demographics, amputation, and prosthetic information of participants fitted with the instrumented prosthesis (i.e., Power Knee, Pro-Flex LP, Pro-Flex XC, Balance S). **BMI:** Body mass index; **TR:** Trauma; **TU:** Tumor; **L:** Left; **R:** Right; **AMP:** Amputation; **TF-BAP:** Transfemoral bone-anchored prosthesis; **%SND:** Percentage of sound thigh length.

No.	Demographics				Amputation				Length of residuum		Prosthesis	
	Age	Height	Mass^1^	BMI^2^	Cause	Side	Time since AMP	Time since TF-BAP			Foot	Footwear
	(Yrs)	(m)	(kg)	(kg/m^2^)		(L/R)	(Yrs)	(Yrs)	(cm)	(%SND)		
1	60	1.77	83	25	TR	R	1.94	1.91	38	87	Pro-Flex LP	Running shoes
2	62	1.78	62	18	TU	R	4.11	3.88	22	50	Pro-Flex LP	Running shoes
3	66	1.83	108	31	TR	R	30.63	9.70	34	77	Pro-Flex LP	Running shoes
4	59	1.78	95	28	TR	R	5.43	4.03	38	77	Pro-Flex LP	Dressing shoes
5	64	1.70	96	33	TR	R	21.06	12.30	28	70	Pro-Flex LP	Running shoes
6	85	1.83	115	33	TR	R	18.73	5.19	32	71	Pro-Flex LP	Flat Shoes
7	56	1.83	95	27	TR	R	3.08	3.06	41	82	Pro-Flex LP	Runners
8	63	1.85	114	32	TR	L	5.93	5.64	38	79	Pro-Flex XC	City Shoes
9	35	1.87	108	29	TR	L	12.39	10.64	32	64	Pro-Flex XC	Runners
10	62	1.83	86	24	TR	L	9.44	5.23	33	62	Pro-Flex XC	Flat shoes
11	81	1.67	73	25	TR	R	7.02	3.89	35	70	Pro-Flex XC	Runners
12	59	1.86	97	27	TU	L	20.15	9.31	34	64	Balance S	Running shoe
13	76	1.73	86	27	TR	L	2.46	2.46	32	68	Balance S	Trekking shoes
**Mean**	**64**	**1.79**	**93.6**	**27.6**			**10.95**	**5.94**	**33.6**	**71.0**		
**SD**	**13**	**0.06**	**15.5**	**4.2**			**9.00**	**3.39**	**4.9**	**9.8**		

1 Body mass without prosthesis;

2 Calculated based on body mass without prosthesis.

A total of 1,327 GCs was analyzed including 538 for walking, 230 for ascending ramps, 265 for descending ramp, 137 for ascending stairs and 157 for descending stairs activities (**Appendix-Table S4**). Only 7 (54%) participants could perform stairs activities using “step-over-step technique (e.g., two-stairs at the time) and “foot on the edge of the step” techniques.

### Spatiotemporal Gait Variables

As detailed in **Table 3-A**, 10 (67%) spatiotemporal variables showed a low variability across all activities. However, high variability was noticeable for five (33%) variables including the cadence during walking and descending ramp as well as the duration of the GC during walking, descending a ramp and ascending stairs.

**Table 3:** Mean and standard deviation as well as variability of spatiotemporal variables, loading boundaries and loading extrema applied on the instrumented prosthesis with the Power Knee. **SD:** Standard deviation; **S:** Second; **%GC:** Percentage of gait cycle; **F:** Force; **M:** Moment; **LG:** Long axis; **AP:** Anteroposterior axis; **ML:** Mediolateral axis; **%BW:** Percentage of the bodyweight; **%SUP:** Percentage of the support phase; **H:** High percentage of variation; **L:** Low percentage of variation; **FLG1:** First point of interest of the force applied on the long axis; **FAP1:** First point of interest of the force applied on the anteroposterior axis; **FML1:** First point of interest of the force applied on the mediolateral axis; **MLG1:** First point of interest of the moment applied around on the long axis; **MAP1:** First point of interest of the moment applied around the anteroposterior axis; **MML1:** First point of interest of the moment applied around the mediolateral axis; **FAP2:** Second point of interest of the force applied on the anteroposterior axis; **MLG2:** Second point of interest of the moment applied around on the long axis; **MML2:** Second point of interest of the moment applied around the mediolateral axis; **MML3:** Third point of interest of the moment applied around mediolateral axis.

	Walking		Ascending ramp		Descending ramp		Ascending stairs		Descending stairs	
**A-Spatiotemporal variables**										
Cadence (Strides/min)	49 ± 13	H	46 ± 8	L	43 ± 10	H	34 ± 6	L	45 ± 6	L
Gait cycle (s)	1.3 ± 0.3	H	1.3 ± 0.2	L	1.5 ± 0.4	H	1.9 ± 0.4	H	1.4 ± 0.2	L
Support (%GC)	63 ± 5	L	63 ± 4	L	62 ± 7	L	58 ± 6	L	52 ± 6	L
**B-Loading boundaries**										
**Minimum**										
FLG (%BW)	-0.9 ± 2.0	H	-0.5 ± 0.7	H	-0.4 ± 0.9	H	-4.4 ± 3.5	H	-2.7 ± 6.8	H
FAP (%BW)	-10.1 ± 3.8	H	-8.8 ± 3.4	H	-14.8 ± 6.2	H	-19.9 ± 6.4	H	-28.2 ± 7.4	H
FML (%BW)	-1.1 ± 1.1	H	-1.1 ± 1.4	H	-0.7 ± 0.6	H	-0.4 ± 0.6	H	-1.1 ± 1.0	H
MLG (%BWm)	-0.53 ± 0.33	H	-0.33 ± 0.26	H	-0.61 ± 0.41	H	-0.81 ± 0.28	H	-0.95 ± 0.41	H
MAP (%BWm)	-3.61 ± 1.07	H	-3.50 ± 1.04	H	-3.07 ± 1.10	H	-2.92 ± 0.61	H	-2.34 ± 0.70	H
MML (%BWm)	-2.32 ± 0.70	H	-2.14 ± 0.62	H	-3.58 ± 1.89	H	-3.20 ± 1.05	H	-6.14 ± 1.06	L
**Maximum**										
FLG (%BW)	102.4 ± 7.1	L	100.4 ± 4.1	L	99.6 ± 11.4	L	99.4 ± 5.4	L	84.1 ± 14.8	L
FAP (%BW)	16.4 ± 4.9	H	16.4 ± 3.3	H	8.6 ± 5.7	H	6.8 ± 4.6	H	4.3 ± 1.7	H
FML (%BW)	10.1 ± 3.7	H	9.7 ± 3.6	H	8.5 ± 2.9	H	8.8 ± 3.7	H	6.3 ± 2.9	H
MLG (%BWm)	0.88 ± 0.44	H	0.97 ± 0.34	H	0.34 ± 0.32	H	0.81 ± 0.36	H	0.21 ± 0.16	H
MAP (%BWm)	0.61 ± 0.42	H	0.64 ± 0.49	H	0.37 ± 0.23	H	0.66 ± 0.41	H	0.44 ± 0.25	H
MML (%BWm)	3.41 ± 1.29	H	5.11 ± 1.17	H	1.70 ± 1.28	H	6.19 ± 1.90	H	0.63 ± 0.35	H
**C-Loading extrema**										
**Onset**										
**Weight acceptance**										
FLG1 (%SUP)	41.8 ± 14.63	H	50.3 ± 15.8	H	38.5 ± 13.8	H	73.6 ± 17.6	H	21.8 ± 12.8	H
FAP1 (%SUP)	16.9 ± 6.2	H	17.6 ± 6.2	H	40.5 ± 26.0	H	18.8 ± 9.9	H	56.2 ± 17.2	H
FML1 (%SUP)	44.2 ± 12.8	H	44.0 ± 11.9	H	45.6 ± 13.4	H	67.7 ± 20.3	H	30.9 ± 14.4	H
MLG1 (%SUP)	23.7 ± 11.3	H	15.7 ± 7.0	H	35.7 ± 21.4	H	19.1 ± 7.7	H	53.4 ± 17.1	H
MAP1 (%SUP)	45.0 ± 14.0	H	45.0 ± 13.6	H	47.8 ± 14.7	H	65.2 ± 25.2	H	26.3 ± 12.9	H
MML1 (%SUP)	13.6 ± 11.0	H	6.5 ± 5.8	H	35.0 ± 31.3	H	52.8 ± 23.3	H	67.6 ± 14.3	H
**Propelling loads**										
FAP2 (%SUP)	79.2 ± 5.1	L	79.1 ± 5.0	L	88.8 ± 9.9	L	76.6 ± 25.6	H	-	-
MLG2 (%SUP)	68.6 ± 11.0	L	61.8 ± 11.7	L	77.9 ± 21.8	H	69.8 ± 20.8	H	-	-
MML2 (%SUP)	65.2 ± 9.2	L	62.3 ± 8.9	L	83.5 ± 8.7	L	-	-	-	-
MML3 (%SUP)	91.8 ± 5.4	L	92.3 ± 4.9	L	-	-	-	-	-	-
**Magnitude**										
**Weight acceptance**										
FLG1 (%BW)	102.4 ± 7.1	L	100.4±4.0	L	99.6±11.4	L	99.4±5.4	L	84.1±14.8	L
FAP1 (%BW)	-10.1±3.8	H	-8.8±3.4	H	-14.7±6.1	H	-19.9±6.4	H	-28.2±7.4	H
FML1 (%BW)	10.1±3.7	H	9.7±3.6	H	8.5±2.9	H	8.8±3.7	H	6.3±2.9	H
MLG1 (%BWm)	-0.53±0.34	H	-0.31±0.27	H	-0.59±0.42	H	-0.81±0.28	H	-0.94±0.41	H
MAP1 (%BWm)	-3.61±1.07	H	-3.50±1.04	H	-3.07±1.10	H	-2.92±0.61	H	-2.34±0.70	H
MML1 (%BWm)	-0.85±1.02	H	-0.26±0.43	H	1.70±1.28	H	6.19±1.90	H	-6.14±1.06	L
**Propelling loads**										
FAP2 (%BW)	16.4±4.9	H	16.4±3.3	H	8.2±6.2	H	6.6±4.7	H	-	-
MLG2 (%BWm)	0.88±0.44	H	0.97±0.34	H	0.30±0.35	H	0.80±0.38	H	-	-
MML2 (%BWm)	3.32±1.37	H	5.01±1.31	H	-3.48±1.93	H	-	-	-	-
MML3 (%BWm)	-2.27±0.64	H	-2.14±0.63	H	-	-	-	-	-	-

**Figure 1:**
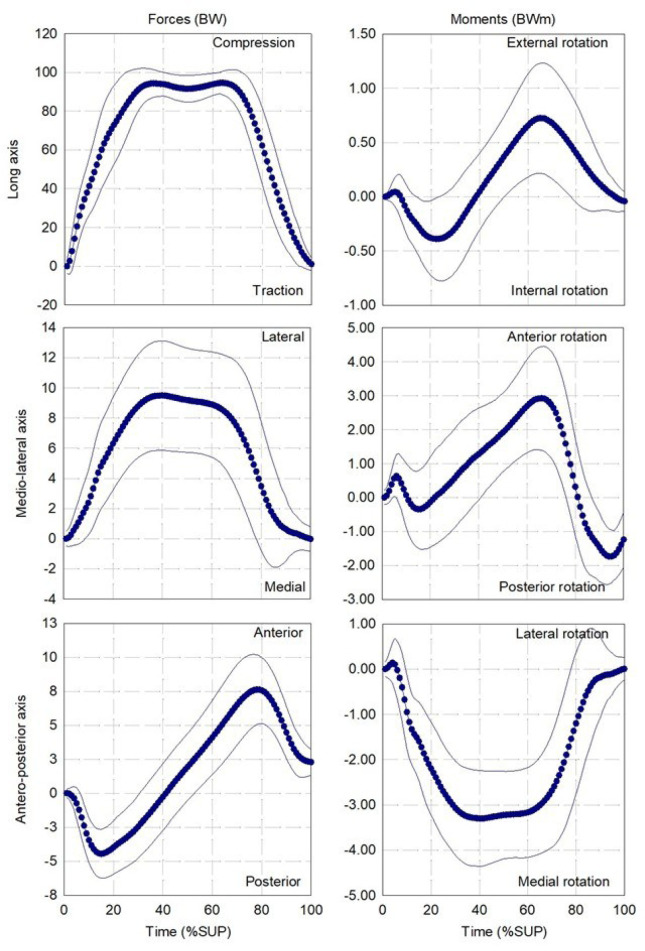
Average and standard deviation (thin lines) of loading profile applied on instrumented prosthesis with the Power Knee during walking (13 participants, 538 gait cycles). **%BW:** Percentage of the bodyweight; **%SUP:** Percentage of the support phase.

**Figure 2:**
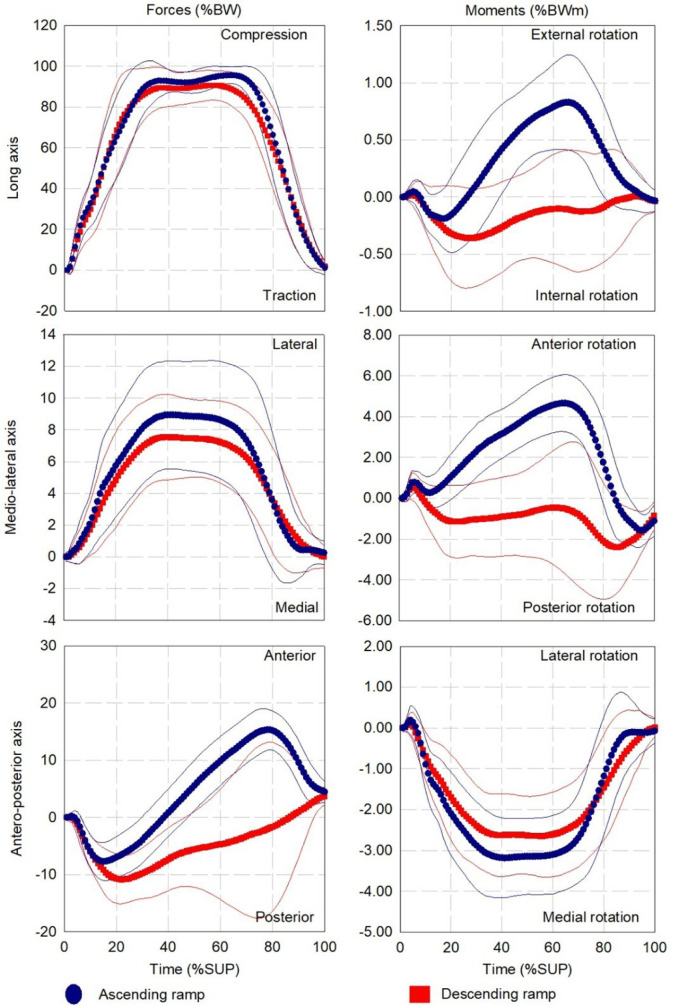
Average and standard deviation (thin lines) of loading profile applied on the instrumented prosthesis with the Power Knee during ascending (12 participants, 230 gait cycles) and descending (12 participants, 265 gait cycles) ramp. **%BW:** Percentage of the bodyweight; **%SUP:** Percentage of the support phase.

**Figure 3:**
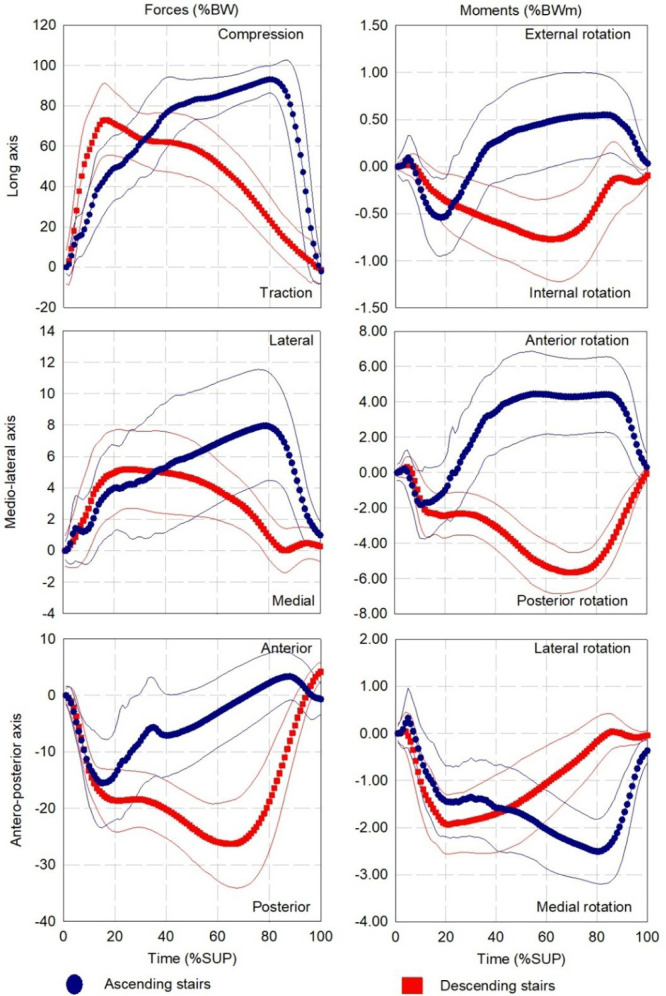
Average and standard deviation (thin lines) of loading profile applied on the instrumented prosthesis with the Power Knee during ascending (7 participants, 137 gait cycles) and descending (7 participants, 157 gait cycles) stairs. **%BW:** Percentage of the bodyweight; **%SUP:** Percentage of the support phase.

The percutaneous part was 0.8 ± 1.7 cm, -0.1 ± 0.6 cm and 9.6 ± 1.5 cm while the geometrical center of the Power Knee was 0.1 ±1.3 cm, -0.2 ± 0.9 cm and -8.4 ± 0.6 cm away from the center of the transducer on the AP, ML and LG axes, respectively (**Appendix-Figure S3, Table S2, Figure S4, Figure S5**). The mean and standard deviation of the loading pattern applied on the transducer over the support phase during walking, ascending and descending ramp and stairs are presented in **Figure 1, Figure 2** and **Figure 3**, respectively.

### Loading Boundaries

As presented in **Table 3-B**, 54 (90%) out of the 60 loading boundaries showed high variability. The variability was low for the average minimum load on MML during ascending stairs and the average maximum loads on FLG in all activities. The loading ranged between:

-305 N or -32 %BW and 1,258 N or 147 %BW on FLG,
-331 N or -47 %BW and 224 N or 25 %BW on FAP,
-47 N or -6 %BW and 234 N or 21 %BW on FML,
- 17 Nm or -2.2 %BWm and 19 Nm or 2.0 %BWm on MLG,
-74 Nm or -6.6 %BWm and 20 Nm or 1.9 %BWm on MAP,
-82 Nm or -8.7 %BWm and 91 Nm or 9.7 %BWm on MML.

The positive and negative values depended on the orientation of the transducer coordinate system, as presented in **Table 1**-Steps 6 and 7.

### Loading Extrema

**Figure S6:**
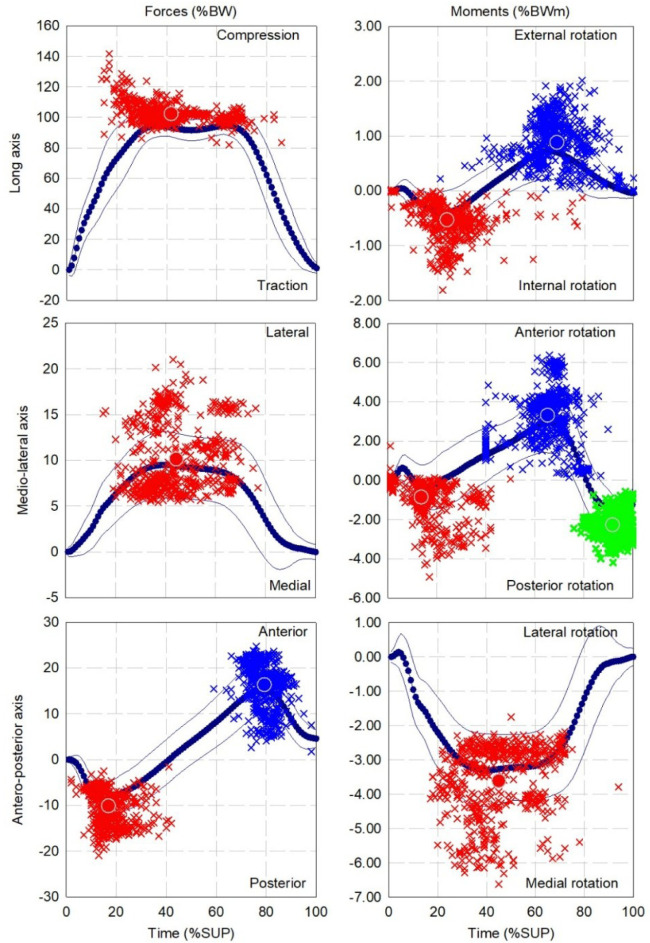
Dispersion (cross) and average (circle) for first (red), second (bleu) and third (green) points of interest of forces and moments for cohort of participants (N=13) fitted instrumented prosthesis (i.e., Power Knee, Pro-Flex LP, Pro-Flex XC, Balance S) during walking (538 gait cycles).

**Figure S15:**
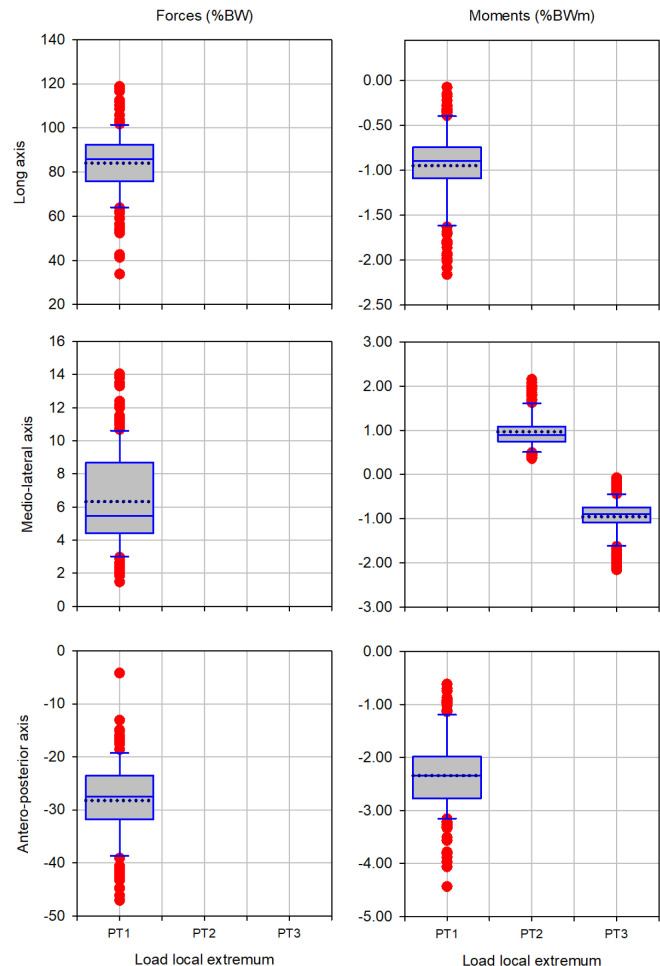
Box plots showing low and high 95% confidence interval, mean and outliers of the magnitude of up to three local extrema (PT1, PT2, PT3) of forces and moments cohort of participants (N=7) fitted with instrumented prosthesis (i.e., Power Knee, Pro-Flex LP, Pro-Flex XC, Balance S) during descending stairs (157 gait cycles).

As detailed in **Table 3-C** and **Appendix-Figure S6-Figure S15**, the loading profile applied during the daily activities was characterized by up to ten extrema for level walking and ascending ramp, nine for descending ramp, eight for ascending stairs and six for descending stairs including:

- First point of interest of the force applied on the long axis (FLG1),
- First point of interest of the force applied on the anteroposterior axis (FAP1),
- Second point of interest of the force applied on the anteroposterior axis (FAP2),
- First point of interest of the force applied on the mediolateral axis (FML1),
- First point of interest of the moment applied around on the long axis (MLG1),
- Second point of interest of the moment applied around on the long axis (MLG2),
- First point of interest of the moment applied around the anteroposterior axis (MAP1),
- First point of interest of the moment applied around the mediolateral axis (MML1),
- Second point of interest of the moment applied around the mediolateral axis (MML2)
- Third point of interest of the moment applied around the mediolateral axis (MML3)^6,26^

Altogether, the onset and magnitude of the extrema showed a high variability for 33 (77%) and 37 (86%) out of 43 extrema, respectively. The six extrema occurring during the weight acceptance phase had an onset and a magnitude with high variability, expected for the magnitude of FLG1 during all activities and MML1 during descending stairs. The four extrema occurring during the propelling phase had an onset with low variability, expected for FAP2 during ascending stairs and MLG2 during descending ramp and ascending stairs, but a magnitude with high variability.

### Benchmark

As presented in **Table 4-A** and **Appendix-Table S5**, the duration of the support phases was also 7 %GC, 11% longer above MCID compared to N-MPKs. The differences in all the other spatiotemporal gait variables between the Power Knee and participants fitted with socket prostheses, N-MPKs and recent P-MPKs were below MCID. The self-selected walking cadence with the Power Knee was 9 strides/min slower than able-bodied participants and 5 strides/min faster than sockets users. The duration of the support phases was also 0.19 s.

**Table 4:** Differences in gait and load characteristics produced with Power Knee compared to reference values produced with Total Knee and Rheo Knee XC during walking.^6,15,16,25,26^

	N-MPK ^(a) 15,16,25^			P-MPK ^(b) 6,26^		
	(Unit)	(%)		(Unit)	(%)	
**A-Spatiotemporal variables**						
Cadence (strides/min)	2.12	4	B	2.43	5	B
Duration gait cycle (s)	-0.02	-1	B	-0.07	-5	B
Duration support (%GC)	6.69	11	A	-0.26	0	B
**B-Loading boundaries** ^(c)^						
FLG (%BW)	16.5	16	A	0.4	0	B
FAP (%BW)	2.5	15	A	-3.4	-21	A
FML (%BW)	-1.0	-10	B	3.1	31	A
MLG (%BWm)	0.40	45	A	0.15	17	A
MAP (%BWm)	0.70	19	A	0.17	5	B
MML (%BWm)	0.89	26	A	-0.72	-21	A
**C-Loading extrema**						
**Weight acceptance**						
FLG1 (%BW)	16.5	16	A	0.4	0.4	B
FAP1 (%BW)	-1.5	15	A	1.3	-12	A
FML1 (%BW)	-1.0	-10	B	3.1	31	A
MLG1 (%BWm)	-0.11	22	A	-0.10	18	A
MAP1 (%BWm)	-0.70	19	A	-0.17	5	B
MML1 (%BWm)	1.00	-117	A	-0.10	12	A
**Propelling loads**						
FAP2 (%BW)	2.5	15	A	-3.4	-21	A
MLG2 (%BWm)	0.40	46	A	0.15	17	A
MML2 (%BWm)	1.87	57	A	-0.78	-23	A
MML3 (%BWm)	0.17	-7	B	0.20	-9	B

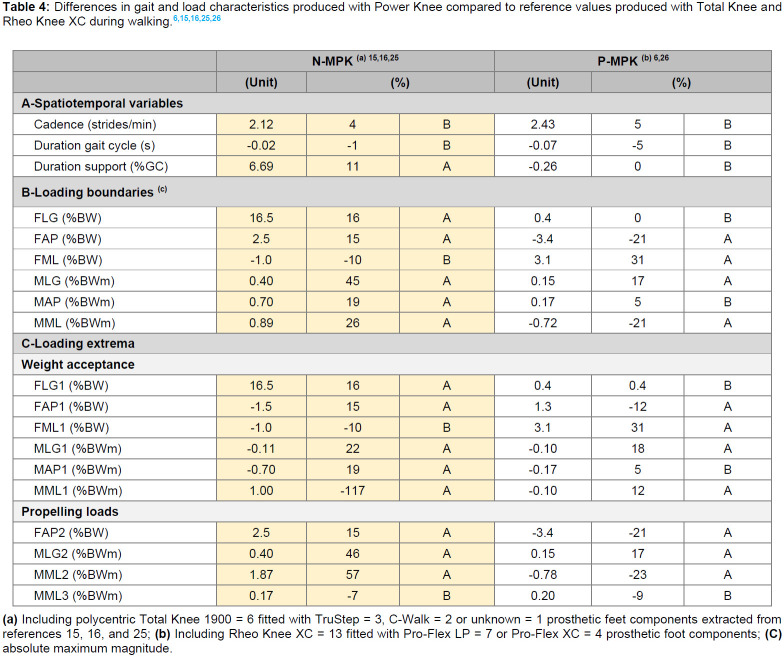

**(a)** Including polycentric Total Knee 1900 = 6 fitted with TruStep = 3, C-Walk = 2 or unknown = 1 prosthetic feet components extracted from references 15, 16, and 25;

**(b)** Including Rheo Knee XC = 13 fitted with Pro-Flex LP = 7 or Pro-Flex XC = 4 prosthetic foot components;

**(c)** absolute maximum magnitude.

As outlined in **Table 4-B** and **Appendix-Table S5-B**, the differences between forces and moments applied by the Power Knee and TF-BAP fitted with N-MPK during walking were ranged between -4 N or -1.00 %BW and 209 N or 16.50 %BW as well as 4.16 Nm or 0.40 %BWm and 9.98 Nm or 0.89 %BWm, respectively. The six differences between forces and moments applied were above MCID expected from FML. Four out of six differences between forces and moments applied on the Power Knee and TF-BAP fitted with P-MPKs were also above MCID ranging between -13 N or -3.38 %BW and 61 N or 3.13 %BW as well as -5 Nm or -0.72 %BWm and 4 Nm or 0.17 %BWm, respectively.

As detailed in **Table 4-C** and **Appendix-Table S5-C**, walking with the Power Knee reduced three extrema (i.e., FAP1, MLG1, MAP1) and increased two extrema (i.e., FLG1, MML1) during the weight acceptance while increasing three extrema (e.g., FAP2, MLG2, MML3) during propelling loading above MCID compared to N-PMK. The Power Knee increased all forces extrema and reduced all moments extrema during the weight acceptance while increasing and decreasing two extrema during propelling loading compared to P-MPK.

## DISCUSSION

This cross-sectional study characterized the loads applied to an instrumented TF-BAP fitted with an A-MPK (Power Knee, Össur, Iceland) during standardized daily activities.

### Key Results

This cross-sectional study showed that the load applied on TF-BAP fitted with a Power Knee was characterized by:

- A self-selected cadence ranging from 34 ± 6 to 49 ± 13 strides/min during walking, ascending and descending ramp and stairs.
- Absolute loading boundaries equal to 147 %BW on FLG, 47 %BW on FAP, 21 %BW on FML, 2.2 %BWm on MLG, 6.6 %BWm on MAP and 9.7 %BWm on MML.
- A low variability for 10 (67%) of spatiotemporal variables but high variability for 54 (90%) loading boundaries and magnitude of 37 (86%) loading extrema.

### Interpretation

Similarly to previous studies, the outcomes showed a high variability typical of symptomatic populations like TFAs.^6,15,16,25,26^ Several factors of heterogeneity might contribute to high variability such as the diversity of prosthetic feet, alignment of the prosthesis and offset of the transducer as well as short acclimation as detailed below.

Compared to reference values collated by Frossard et al,^20,25,52^ the instrumented TF-BAP fitted with the Power Knee may restore noticeably the spatiotemporal gait variables. Indeed, the cadence was 11% faster above MCID than participants fitted with socket, at least when ambulating at self-selected pace. The loading boundaries were found to be within a range considered appropriate for safe coupling between healthy bone and implant.

The extrema occurring during the weight acceptance and propelling loading including FLG1 and FAP2 were 16.50 %BW (16%) and 2.49 %BW (15%) higher and above MCID for the Power Knee compared to TF-BAP fitted with N-MPK, respectively. Further studies will be required to confirm that these increases might translate into more symmetrical loading with the sound limb.^53-55^ Altogether, these outcomes suggest that the Power Knee may contribute to improve the walking ability, particularly walking pace, compared to N-MPK.

The outcomes of the comparison with recent studies involving the Rheo Knee XC and Pro-Flex feet might be less certain. FLG1 was 0.42 %BW (0.41%) higher but below MCID with the Power Knee confirming its weight acceptance abilities. FAP2 was 3.38 %BW (21%) less and above MCID with the Power Knee suggesting a relatively lower capacity to generate propelling forces. However, differences might be due to a larger proportion of participants fitted with Pro-Flex XC (n=9, 70%) in the P-MPK study.^15,16,25^ The design of the Pro-Flex XC allows higher ankle push-off power and range of motion compared to the Pro-Flex LP.^56-58^ Further investigations are required to establish the impact of prosthetic feet varying in stiffness and range of movement on the loading profiles of TF-BAP (e.g., index of anthropomorphy).^12,48,59^

### Limitations

The limitations constricting clinical interpretations inherent to the study design related to: the sample size; the presentation of the load datasets in relation to the transducer rather than the implant; the offset of the transducer attached to the connector; the dynamic alignments without standardization and stiffness of the prosthetic feet; the lack of spatial (e.g., walking base, step and stride length), dynamics (e.g., ground and handrail reaction forces), kinematics (e.g., trunk bending, hip range of movement) and kinetics (e.g., ankle, knee, and hip joint moments and work) characteristics; and, the educated choices for the PV's and MCID's thresholds.

A specific limitation was the short acclimation with the instrumented prosthesis. We anticipated that participants will acclimate rapidly to the instrumented prosthesis given their previous experience with P-MPKs or A-MPKs.^47^ However, optimizing individual settings for each task might required more than one session with the prosthetist and a longer adaptation. So, limited adaptation time to the active support of the device especially during ramp and stair activities might have led to a more tentative and variable gait pattern and slower walking speeds (e.g., increase variability of extrema, decrease propelling loading, use of the handrail).

### Generalization

The main barrier to generalizing these outcomes was the relatively small sample size (N = 13) and the male-dominated cohort. COVID-19 pandemic impeded recruitment of participants and extensive testing (e.g., acclimation). However, our ecological approach allowed to capture a larger number of steps than typical studies relying on fixed-equipment.^21,27-32,53,54,60-63^ As mentioned above, several weeks of acclimation might decrease variability and increase the generalization of the outcomes. The generalization of the outcomes to other commercially available powered knees might be uncertain due to different specificities of their design.

### Future Studies

This study can inform the design of subsequent observational studies with larger cohorts focusing on loading profile applied on TF-BAP with various component configurations (ESARs, P-MPKs, A-MPKs).^55,64-67^ Practically, the range of loading characteristics presented here can facilitate the calculation of cohorts' sample sizes (e.g., statistical power).

The understanding of the benefits of TF-BAP fitted with powered knees can be extended by other studies focusing on: functional outcomes; 3D dynamic, kinematic, kinetic; metabolic characteristics; and participant's experience (e.g., device weight and noise).^17,21,27,67^ Furthermore, there is a need to establish the cause-effect relationships between loading characteristics and confounders related to demographics, amputation history, prosthetic arrangement, walking ability as well as the strength and safety of bone-implant coupling.^61,68-74^ Finally, new systematic reviews and meta-analyses relying on advanced statistical approaches are required to determine the loading variability associated with components currently recommended for TF-BAP as well as their efficacy and safety (e.g., walking pace, weight acceptance, propelling load).^13,73^

## CONCLUSION

Benchmark loading data for a powered knee currently recommended for TF-BAP is provided for the first time. Altogether, the spatiotemporal gait characteristics and the propelling loads suggested that fitting the Power Knee alongside Pro-Flex (XC, LP) and Balance S prosthetic feet may restore distinctly the capacity of participants fitted a transfemoral osseointegrated implant to ambulate. Indicative comparisons with the literature suggested that the loading profile applied with this combination of components is more suitable than N-MPKs and stacked up against recent P-MPKs. Therefore, one can argue that a routine transition from N-MPKs or P-MPKs onto the Power Knee appears safe and potentially effective.

As listed above, this is the third study applying this protocol to assess ecological TF-BAP prosthetic loading. This protocol can facilitate cross-comparison of loading characteristics between studies. However, further standardization requires a consensus around loading criteria likely to warrant efficacy and safety of TF-BAP components (e.g., weight acceptance, propelling loading). These efforts might also contribute to the design of ISO norms for osseointegrated implants and BAP-specific components.

In the meantime, this study producing Level IV evidence, participated in evidence-based prescription of TF-BAP fitted with powered knees. Hopefully, this work will also contribute to the developments of standard of care for growing population of individuals using bionic limbs.

## Figures and Tables

**Figure S7:**
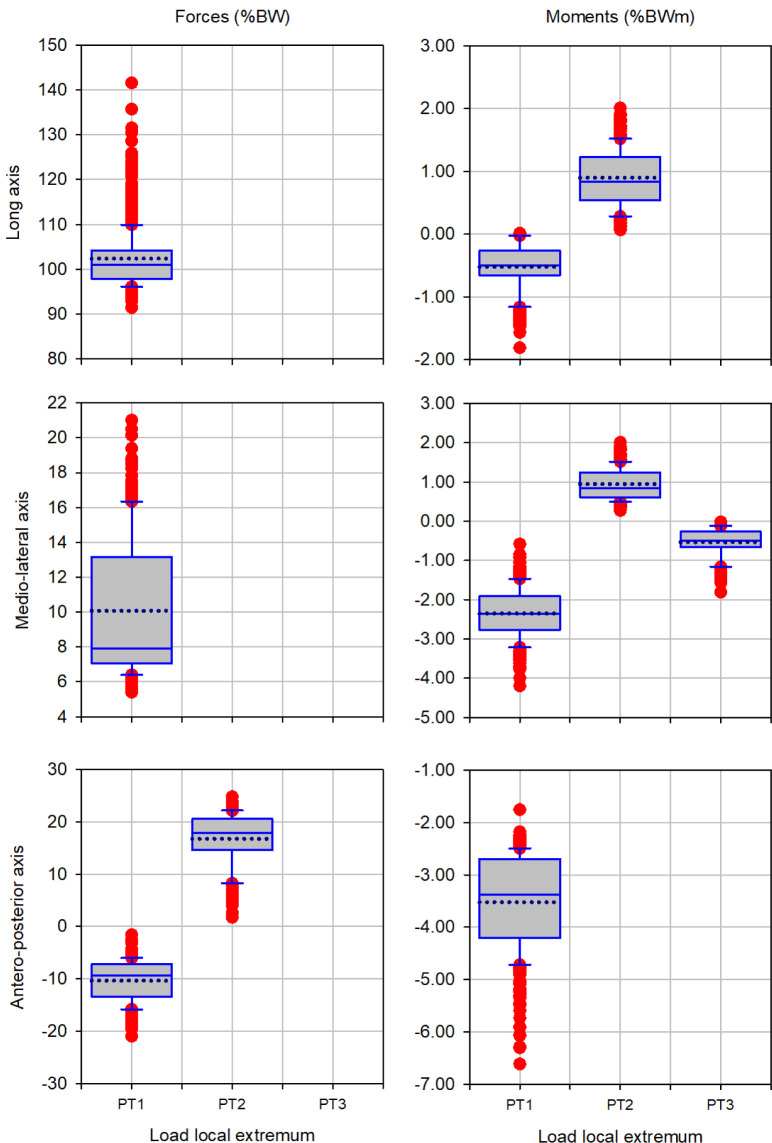
Box plots showing low and high 95% confidence interval, mean and outliers of the magnitude of up to three local extrema (PT1, PT2, PT3) of forces and moments for cohort of participants (N=13) fitted instrumented prosthesis (i.e., Power Knee, Pro-Flex LP, Pro-Flex XC, Balance S) during walking (538 gait cycles).

**Figure S8:**
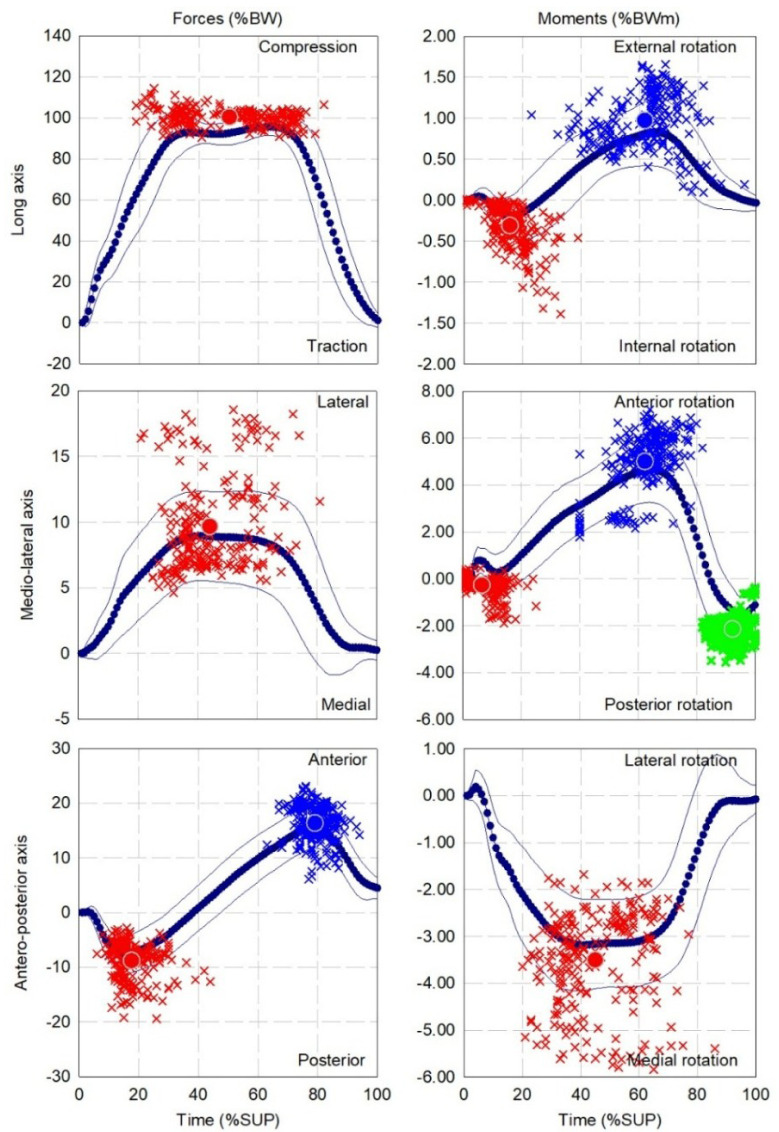
Dispersion (cross) and average (circle) for first (red), second (bleu) and third (green) points of interest of forces and moments for cohort of participants (N=12) fitted with instrumented prosthesis (i.e., Power Knee, Pro-Flex LP, Pro-Flex XC, Balance S) during ascending ramp (230 gait cycles).

**Figure S9:**
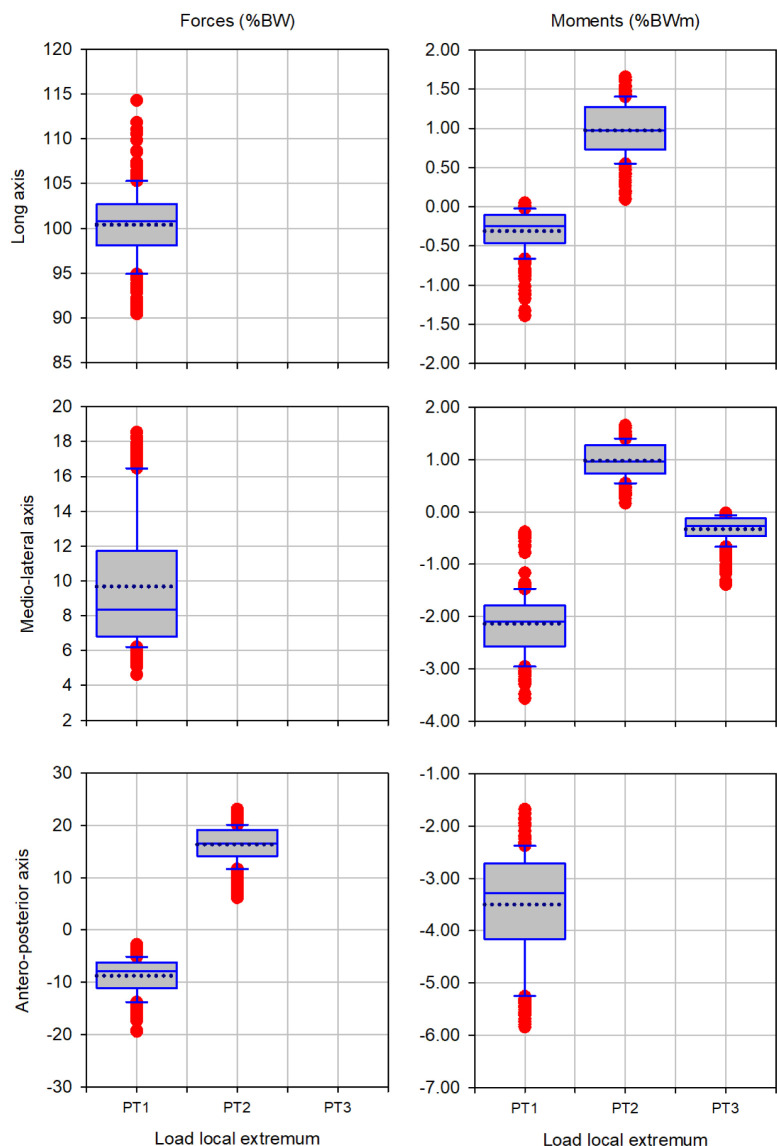
Box plots showing low and high 95% confidence interval, mean and outliers of the magnitude of up to three local extrema (PT1, PT2, PT3) of forces and moments for cohort of participants (N=12) fitted with instrumented prosthesis (i.e., Power Knee, Pro-Flex LP, Pro-Flex XC, Balance S) during ascending ramp (230 gait cycles).

**Figure S10:**
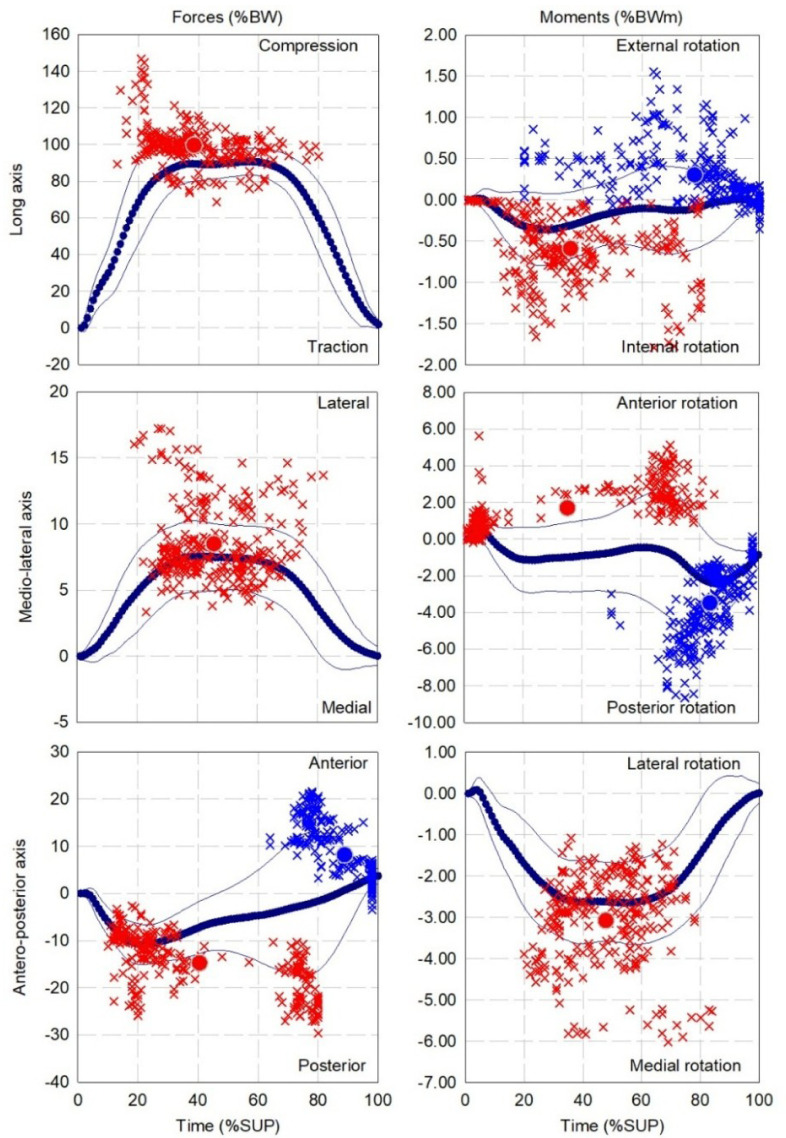
Dispersion (cross) and average (circle) for first (red), second (bleu) and third (green) points of interest of forces and moments for cohort of participants (N=12) fitted with instrumented prosthesis (i.e., Power Knee, Pro-Flex LP, Pro-Flex XC, Balance S) during descending ramp (265 gait cycles).

**Figure S11:**
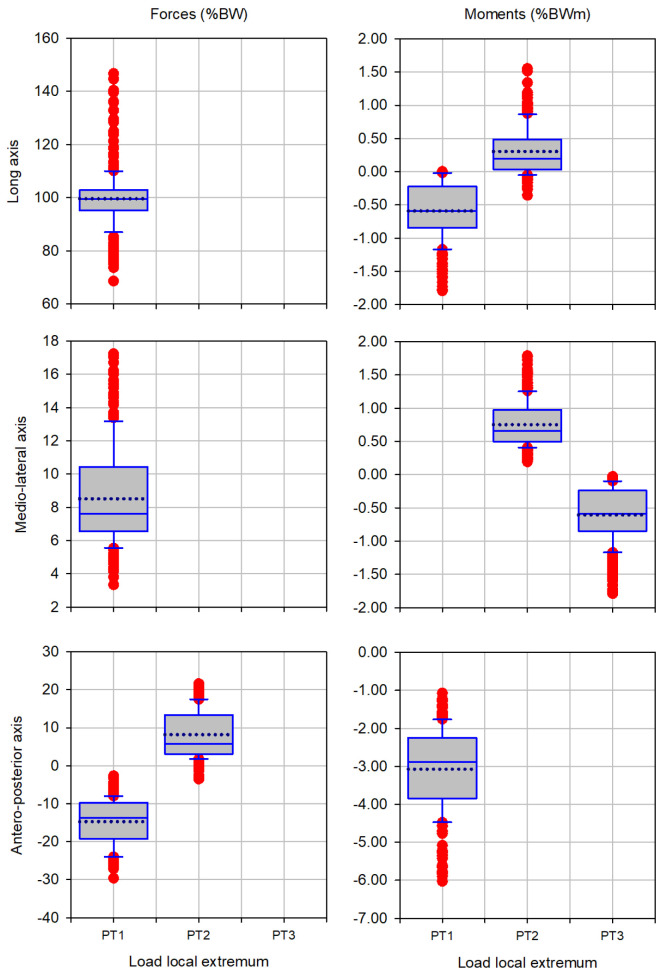
Box plots showing low and high 95% confidence interval, mean and outliers of the magnitude of up to three local extrema (PT1, PT2, PT3) of forces and moments for cohort of participants (N=12) fitted with instrumented prosthesis (i.e., Power Knee, Pro-Flex LP, Pro-Flex XC, Balance S) during descending ramp (265 gait cycles).

**Figure S12:**
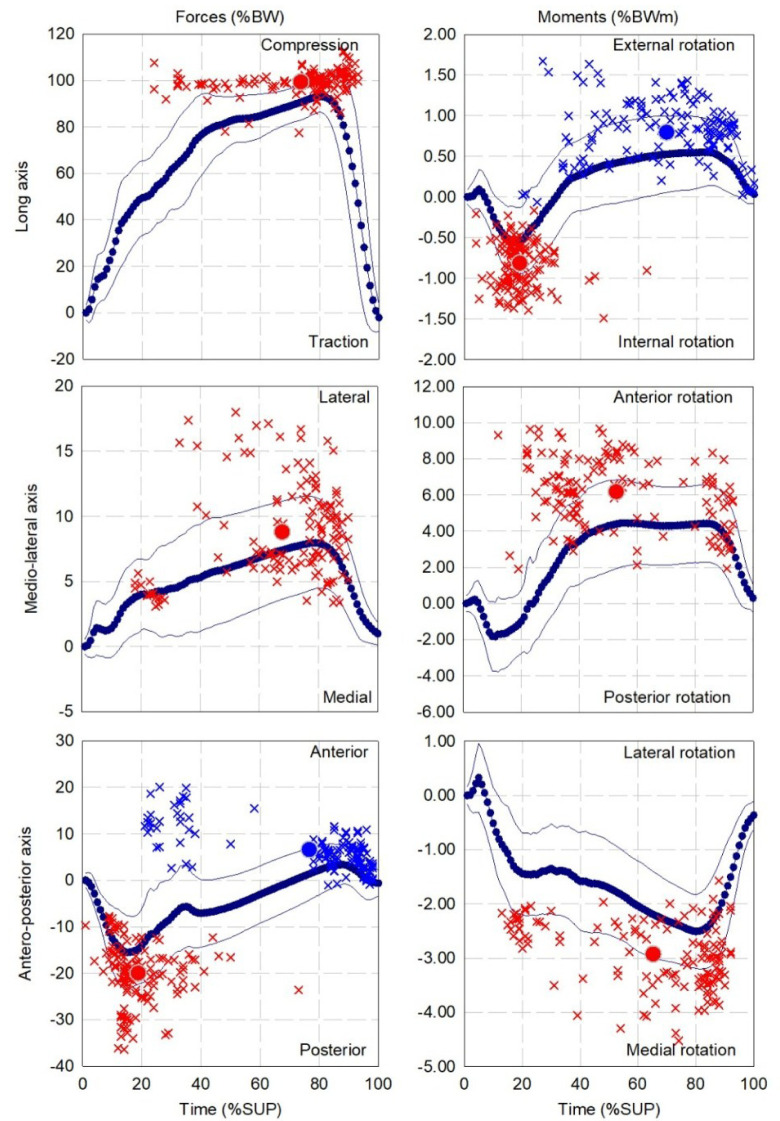
Dispersion (cross) and average (circle) for first (red), second (bleu) and third (green) points of interest of forces and moments for cohort of participants (N=7) fitted with instrumented prosthesis (i.e., Power Knee, Pro-Flex LP, Pro-Flex XC, Balance S) during ascending stairs (137 gait cycles.

**Figure S13:**
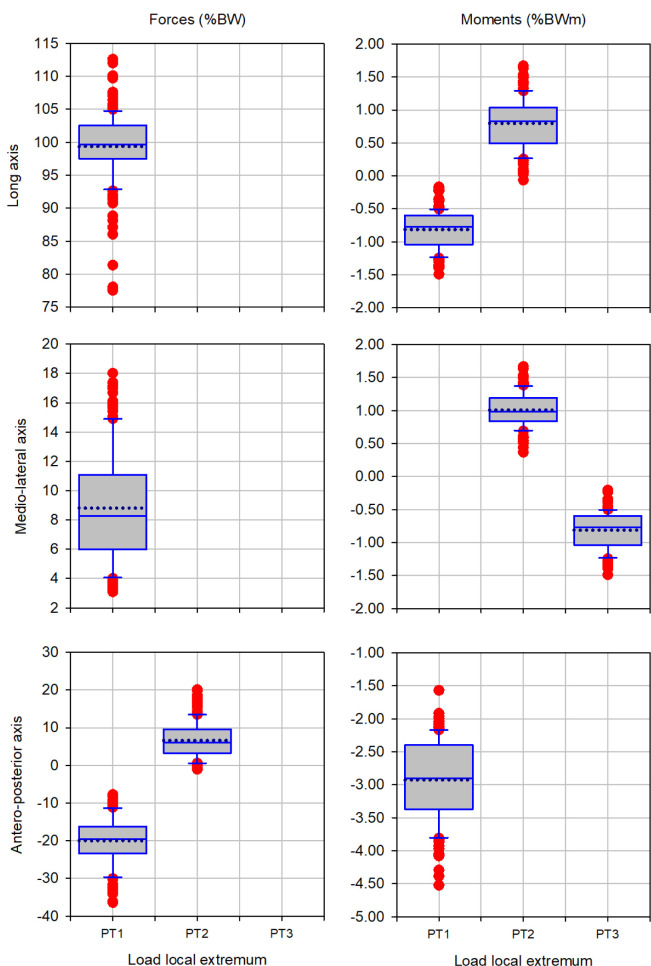
Box plots showing low and high 95% confidence interval, mean and outliers of the magnitude of up to three local extrema (PT1, PT2, PT3) of forces and moments for cohort of participants (N=7) fitted with instrumented prosthesis (i.e., Power Knee, Pro-Flex LP, Pro-Flex XC, Balance S) during ascending stairs (137 gait cycles).

**Figure S14:**
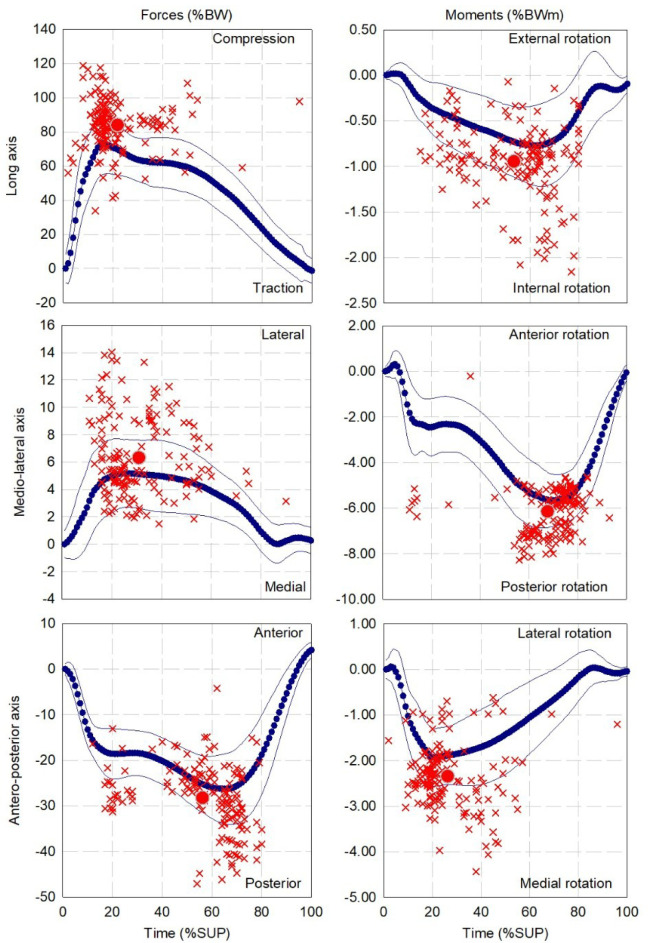
Dispersion (cross) and average (circle) for first (red), second (bleu) and third (green) points of interest of forces and moments for cohort of participants (N=7) fitted with instrumented prosthesis (i.e., Power Knee, Pro-Flex LP, Pro-Flex XC, Balance S) during descending stairs (157 gait cycles).

## APPENDIX

The supplement provides information about the confounders (e.g., selection criteria, alignment of instrumented prostheses, position of the percutaneous part and prosthetic knee in relation to the transducer, setup, number of steps analyzed), the dispersion and magnitude of extrema for each activity as well as comparative values for demographics, spatiotemporal variables, loading boundaries and loading extrema extracted from the literature.

### Confounders

**Table S1:** Selection criteria applied for the recruitment of participants with unilateral transfemoral bone-anchored prosthesis.

**A-Inclusion criteria**	
**1**.	To be willing to participate to this project of research
**2**.	To be willing to comply with protocol
**3**.	To be between 18–80 years of age
**4**.	To be fitted with osseointegrated fixation more than 6 months prior testing
**5**.	To be fully rehabilitated
**6**.	To be able to walk 200 meters independently with prosthesis
**7**.	To be able to be fitted with the nominated ÖSSUR components
**8**.	To be a previous or current user of microprocessor-controlled knee
**9**.	To have a clearance of at least 6–8 cm between connector attached to distal end of percutaneous part of the fixation and prosthetic knee joint to fit the transducer
**B-Exclusion criteria**	
**1**.	To not be able to give informed consent
**2**.	To have mental illness or intellectual impairment
**3**.	To have major uncorrected visual deficit
**4**.	To have history of epilepsy or recurrent dizziness
**5**.	To have bilateral amputation
**6**.	To have self-reported pain level greater than 4 out of 10 at study outset
**7**.	To have experienced a fall within the last 8 weeks before assessment
**8**.	To present signs of infection 2 weeks prior testing session
**9**.	To have injuries involving contralateral (intact) limb

**Table S2:** Position of the distal end of connector attached to the percutaneous part and the geometrical centre of the Power Knee in relation to the origin of coordinate system of iPecsLab's transducer (RTC Electronics, USA) on the antero-posterior (AP), medio-lateral (ML) and vertical (VT) axes.

Participant	Distal end of the percutaneous part			Centre of the Power Knee		
	AP	ML	VT	AP	ML	VT
	(cm)	(cm)	(cm)	(cm)	(cm)	(cm)
1	0.63	-0.46	9.19	-0.67	-0.36	-8.20
2	2.19	0.72	9.91	-1.85	-1.33	-9.93
3	-0.22	0.00	9.08	-0.79	-0.59	-8.83
4	0.77	-1.20	8.12	0.39	1.16	-8.14
5	-0.61	0.01	8.86	-1.06	-0.85	-8.08
6	3.80	-0.59	8.23	-0.51	0.15	-8.56
7	0.63	-0.73	8.77	1.41	1.19	-8.60
8	-1.29	0.22	9.92	2.66	-0.81	-7.70
9	-2.02	0.72	8.69	-0.83	-1.13	-8.36
10	-0.87	0.16	12.08	-0.43	-0.82	-8.74
11	1.65	-0.44	8.59	1.29	0.54	-8.54
12	2.67	0.09	9.79	1.72	0.69	-7.84
13	2.52	0.16	13.41	0.33	-0.23	-7.79
**Mean**	**0.76**	**-0.10**	**9.59**	**0.13**	**-0.18**	**-8.41**
**SD**	**1.74**	**0.56**	**1.54**	**1.31**	**0.85**	**0.58**

**Table S3:** Description of non-experimental facilities.

Activities	Power Knee
**Straight level walking**	
Location	Indoor
Length (m)	14
**Ascending and descending ramp**	
Location	Indoor
Length (m)	5.70
Incline (deg)	3.72
Handrail height (m)	0.93
**Ascending and descending stairs**	
Location	Indoor
Number of steps	10
Step height (cm)	17
Step depth (cm)	29.5
Step width (cm)	1,130
Handrail height (m)	1

**Table S4:** Overview of number of participants (N) and gait cycles (GC) analyzed during the assessment with the instrumented prosthesis (i.e., Power Knee, Pro-Flex LP, Pro-Flex XC, Balance S).

Activity	Participation	Number of gait cycles
	(N, (%))	(GC)
Level walking	13 (100%)	538
Ascending ramp	12 (92%)	230
Descending ramp	12 (92%)	265
Ascending stairs	7 (54%)	137
Descending stairs	7 (54%)	157
**Total**	**13 (100%)**	**1,327**

### Level walking

**Detection of local extrema**

**Characteristics of local extrema**

### Ascending ramp

**Detection of local extrema**

**Characteristics of local extrema**

### Ascending ramp

**Detection of local extrema**

**Characteristics of local extrema**

### Ascending stairs

**Detection of local extrema**

**Characteristics of local extrema**

### Descending stairs

**Detection of local extrema**

**Characteristics of local extrema**

### Comparative values

**Table S5:** Mean and standard deviation of gait and load characteristics produced with a non-microprocessor-controlled knee (N-MPK) Total Knee and passive microprocessor-controlled knee (P-MPK) Rheo Knee XC during walking and the active microprocessor-controlled knee (A-MPK) Power Knee. SD: Standard deviation, N: number of participants, BMI: body mass index, LoR: length of residuum, %SND: Percentage of sound thigh length, s: Second, %GC: Percentage of gait cycle, F: Force, M: Moment, LG: Long axis, AP: anteroposterior axis, ML: Mediolateral axis, %BW: Percentage of bodyweight.

	**N-MPK** (a) 15,16,25	**P-MPK** (b) 6, 26	**Power Knee** (c)
	(Mean±SD)	(Mean±SD)	(Mean±SD)
**A-Population**			
**Demographics**			
Participants (n)	6	13	13
Male (n)	2	11	13
Female (n)	4	2	0
Age (Yrs)	51 ± 6	57±14	64 ± 13
Height (m)	1.75 ± 0.20	1.78 ± 0.08	1.79 ± 0.06
Mass (kg)	75.94 ± 16.94	86.31 ± 18.03	93.65 ± 15.54
BMI (kg/m^2^)	23.38 ± 2.70	25.92 ± 4.73	27.63 ± 4.18
**Amputation**			
Cause			
Trauma (n)	4	9	11
Tumor (n)	1	2	2
Infection (n)	0	2	0
Other (n)	1	0	0
Left (N)	2	5	5
Right (N)	4	8	8
Time since AMP (Yrs)	28 ± 17	17 ± 19	11 ± 9
Time since BAP (Yrs)	5 ± 2	2 ± 2	6 ± 3
LoR (cm)	20.36 ± 4.87	28.38 ± 5.69	33.62 ± 4.91
LoR (%SND)	49 ± 8	63 ± 11	71 ± 10
**B-Spatio-temporal variables**			
Cadence (strides/min)	47 ± 4	47 ± 6	49 ± 13
Duration gait cycle (s)	1.29 ± 0.11	1.34 ± 0.22	1.27 ± 0.31
Duration support (%GC)	56 ± 2	63 ± 4	63 ± 5
**C-Loading boundaries** (d)			
FLG (%BW)	86 ± 6	102 ± 7	102 ± 7
FAP (%BW)	14 ± 4	20 ± 7	16 ± 5
FML (%BW)	11 ± 4	7 ± 3	10 ± 4
MLG (%BWm)	0.48 ± 0.26	0.73 ± 0.33	0.88 ± 0.44
MAP (%BWm)	2.91 ± 0.87	3.44 ± 0.98	3.61 ± 1.07
MML (%BWm)	2.52 ± 0.93	4.13 ± 1.21	3.41 ± 1.29
**D-Loading extrema**			
**Weight acceptance**			
FLG1 (%BW)	86 ± 6	102 ± 7	102 ± 7
FAP1 (%BW)	-9 ± 4	-11 ± 4	-10 ± 4
FML1 (%BW)	11 ± 4	7 ± 3	10 ± 4
MLG1 (%BWm)	-0.41 ± 0.22	-0.43 ± 0.29	-0.53 ± 0.34
MAP1 (%BWm)	-2.91 ± 0.87	-3.44 ± 0.98	-3.61 ± 1.07
MML1 (%BWm)	-1.85 ± 0.42	-0.75 ± 0.68	-0.85 ± 1.03
**Propelling loads**			
FAP2 (%BW)	14 ± 2	20 ± 7	16 ± 5
MLG2 (%BWm)	0.48 ± 0.26	0.73 ± 0.33	0.88 ± 0.44
MML2 (%BWm)	1.44 ± 1.05	4.10 ± 1.25	3.32 ± 1.37
MML3 (%BWm)	-2.43 ± 0.72	-2.47 ± 1.01	-2.27 ± 0.64

(a) Including polycentric Total Knee 1900 = 6 fitted with TruStep = 3, C-Walk = 2 or unknown = 1 prosthetic foot components extracted from 15,16,25

(b) Including Rheo Knee XC = 13 fitted with Pro-Flex LP = 7 or Pro-Flex XC = 4 prosthetic foot components;

(c) including Power Knee = 13; Pro-Flex LP = 7, Pro-Flex XC = 4, or Balance S = 2 prosthetic foot components;

(d) absolute maximum magnitude.
